# Thermogravimetric analysis, kinetic study, and pyrolysis–GC/MS analysis of 1,1ʹ-azobis-1,2,3-triazole and 4,4ʹ-azobis-1,2,4-triazole

**DOI:** 10.1186/s13065-018-0381-x

**Published:** 2018-03-01

**Authors:** Chenhui Jia, Yuchuan Li, Shujuan Zhang, Teng Fei, Siping Pang

**Affiliations:** School of Materials Science and Engineering, Beijing Institute of Technology, Beijing, 100081 China

**Keywords:** 1,1ʹ-Azobis-1,2,3-triazole, 4,4ʹ-Azobis-1,2,4-triazole, Thermal decomposition, Kinetic study, Thermogravimetric–differential scanning calorimetry (TG–DSC), Pyrolysis–gas chromatography/mass spectrometry (PY–GC/MS)

## Abstract

**Background:**

In general, the greater the number of directly linked nitrogen atoms in a molecule, the better its energetic performance, while the stability will be accordingly lower. But 1,1ʹ-azobis-1,2,3-triazole (**1**) and 4,4ʹ-azobis-1,2,4-triazole (**2**) show remarkable properties, such as high enthalpies of formation, high melting points, and relatively high stabilities. In order to rationalize this unexpected behavior of the two compounds, it is necessary to study their thermal decompositions and pyrolyses. Although a great deal of research has been focused on the synthesis and characterization of energetic materials with **1** and **2** as the backbone, a complete report on their fundamental thermodynamic parameters and thermal decomposition properties has not been published.

**Methods:**

Thermogravimetric–differential scanning calorimetry were used to obtain the thermal decomposition data of the title compounds. Kissinger and Ozawa–Doyle methods, the two selected non-isothermal methods, are presented for analysis of the solid-state kinetic data. Pyrolysis–gas chromatography/mass spectrometry was used to study the pyrolysis process of the title compounds.

**Results:**

The DSC curves show that the thermal decompositions of **1** and **2** are at different heating rates involved a single exothermic process. The TG curves provide insight into the total weight losses from the compounds associated with this process. At different pyrolysis temperatures, the compositions and types of the pyrolysis products differ greatly and the pyrolysis reaction at 500 °C is more thorough than 400 °C.

**Conclusions:**

Apparent activation energies (*E*) and pre-exponential factors (ln*A*/s^−1^) are 291.4 kJ mol^−1^ and 75.53 for **1**; 396.2 kJ mol^−1^ and 80.98 for **2** (Kissinger). The values of *E* are 284.5 kJ mol^−1^ for **1** and 386.1 kJ mol^−1^ for **2** (Ozawa–Doyle). The critical temperature of thermal explosion (*T*_*b*_) is evaluated as 187.01 °C for **1** and 282.78 °C for **2**. The title compounds were broken into small fragment ions under the pyrolysis conditions, which then might undergo a multitude of collisions and numerous other reactions, resulting in the formation of C_2_N_2_ (*m*/*z* 52), etc., before being analyzed by the GC/MS system.

**Electronic supplementary material:**

The online version of this article (10.1186/s13065-018-0381-x) contains supplementary material, which is available to authorized users.

## Background

Triazoles are a class of typical nitrogen-rich compounds, which have been widely used in novel energetic materials, medicine, catalysis, and other fields [1–4]. Over several decades, many studies have shown that azobis-triazole compounds have good energetic properties. This is due to their structure with multiple nitrogen atoms linked directly, along with many C–N and N–N single or double bonds in the molecule, which have good energetic properties [5–11]. Indeed, compared with a single triazole ring, energetic properties such as energy density, heat of formation, detonation velocity, and detonation pressure, can be significantly improved [8, 12–16].

In general, the greater the number of directly linked nitrogen atoms in a molecule, the better its energetic performance, but stability will be accordingly lower [5, 12, 13, 17–22]. The title compounds 1,1ʹ-azobis-1,2,3-triazole (**1**) and 4,4ʹ-azobis-1,2,4-triazole (**2**) show remarkable properties, such as high enthalpies of formation, high melting points, and relatively high stabilities [5, 13, 14, 17]. In order to rationalize this unexpected behavior of the two compounds, it is necessary to study their thermal decompositions and pyrolyses. Although a great deal of research has been focused on the synthesis and characterization of energetic materials with **1** and **2** as the backbone [5, 13, 17, 19], a complete report on their fundamental thermodynamic parameters and thermal decomposition properties has not been published.

The stability of the title compounds can be quantitatively determined by studying their thermodynamic properties, such as apparent activation energy (*E*) and pre-exponential factor (*A*) of thermal decomposition, and the critical temperature of thermal explosion (*T*_*b*_).

There are many methods for analyzing non-isothermal solid-state kinetic data from TG and DSC [23–26]. These methods can be divided into two types: model-fitting and model-free methods, as summarized in Table 1 [27]. Model-fitting methods have been widely used for solid-state reactions because of their ability to directly determine the kinetic parameters from a single TG measurement. However, these methods suffer from several shortcomings, such as their inability to uniquely determine the reaction model, and that application of model-fitting methods to non-isothermal data gives higher values of the kinetic parameters. Conversely, model-free methods require several kinetic curves to perform the analysis. Calculations of several curves at different heating rates are performed on the same value of conversion, which allows calculation of the activation energy at each conversion point. Results from model-free methods tend to be more reliable and reasonable than those from model-fitting methods, especially when dealing with non-thermal data [27].

**Table 1:** Common methods for studying non-isothermal solid-state kinetics

Model-fitting method	Model-free method
Differential	Kissinger
Freeman–Carroll	Kissinger–Akahira–Sunose
Coats–Redfern	Ozawa–Doyle method

Thermal pyrolysis refers to chemical decomposition caused by heat, when the thermal energy applied to the sample exceeding the chemical bond energy of the molecules. PY–GC/MS has been widely used to investigate decomposition processes and pyrolysis products [28]. Analytical pyrolysis allows the thermal breakdown of a molecule into smaller fragments, which are then selected and analyzed by the GC/MS system, providing insight into the decomposition of the sample. The PY–GC/MS can study the thermal pyrolysis of an energetic material and provides information about the nature of the explosion reaction products by GC/MS analysis, which give the information about the reaction process. These in turn can be used to evaluate whether the used explosives are environmental friendly from the identification of the explosive residue species. In addition, the identification of the explosive residue species can also be used in military and counterterrorism practice.

Compound **1** and **2** can be prepared by oxidation of the N–NH_2_ moieties in 1-amino-1,2,3-triazole and 4-amino-1,2,4-triazole, respectively, with sodium dichloroisocyanurate (SDIC). In this study, the thermal decomposition processes of **1** and **2** have been investigated by dynamic TG–DSC under nitrogen atmosphere at different heating rates, and by PY–GC/MS under helium atmosphere at set temperatures of 400 and 500 °C, respectively. Kinetic parameters have been obtained by two model-free methods, the Kissinger method and the Ozawa–Doyle method combined with a kinetic compensation effect. The data reported herein are expected to be of broad interest to researchers engaged in the study and applications of **1** and **2**.

## Materials and methodology

### Materials

**Fig. 1:**
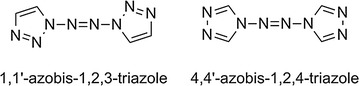
Structures of the title compounds

1,1ʹ-Azobis-1,2,3-triazole (**1**) and 4,4ʹ-azobis-1,2,4-triazole (**2**) were prepared according to literature procedures [13, 14]. The sample purities were > 0.99 (w/w). IR spectra were recorded from solid samples in KBr pellets on a Bruker Tensor 27 spectrometer. Elemental analyses were consistent with the theoretical compositions. The samples were further purified by drying in vacuo at 60 °C for approximately 4 h. The structures of the compounds are shown in Fig. 1.

#### Synthesis of 1,1ʹ-azobis-1,2,3-triazole (**1**) [13]

1-Amino-1,2,3-triazole (1.26 g, 15 mmol) was dissolved in CH_3_CN (40 mL). The solution was cooled to – 5 to 0 °C and a solution of SDIC (3.33 g, 15 mmol) in water (10 mL) and CH_3_COOH (5 mL) was added dropwise. The reaction mixture was further stirred at 0 °C for 30 min. It was then neutralized with NaHCO_3_ and filtered. The filtrate was concentrated to afford the product, which was obtained as a slightly yellow solid after recrystallization from acetone. ^1^H NMR (400 MHz, [D_6_]DMSO, 25 °C, TMS): δ = 9.17 (2H, d), 8.21 ppm (2H, d); ^13^C NMR (100 MHz, [D_6_]DMSO, 25 °C, TMS): δ = 134.8, 118.0 ppm; IR (KBr): ν = 3128, 1625, 1482, 1320, 1225, 1171 cm^−1^; MS: *m*/*z* 165.0 [*M* + H]^+^; elemental analysis calcd. (%) for C_4_H_4_N_8_ (164): C, 29.27; H, 2.44; N, 68.29; found: C, 29.45; H, 2.32; N, 68.23.

#### Synthesis of 4,4ʹ-azobis-1,2,4-triazole (**2**) [14]

Acetic acid (5 mL) was added to a solution of SDIC (5.09 g, 23 mmol) in water (40 mL) with vigorous stirring at 30 °C. After 1 h, the mixture was cooled to 5 °C and a solution of 4-amino-1,2,4-triazole (2.07 g, 25 mmol) in water (10 mL) was added. The reaction mixture was vigorously stirred at 15 °C for 1 h. It was then cooled, and the precipitate that formed was collected by filtration and washed with water at 60 °C. After drying in vacuo, a white product was obtained. ^1^H NMR (400 MHz; D_2_O, 25 °C, TMS): δ = 7.22 ppm (4H, s); ^13^C NMR (100 MHz; D_2_O, 25 °C, TMS): δ = 138.57 ppm; IR (KBr): ν = 3114, 1493, 1368, 1317, 1180 cm^−1^; MS: *m*/*z* 164 (*M*^+^); elemental analysis calcd. (%) for C_4_H_4_N_8_ (164): C, 29.27; H, 2.44; N, 68.29; found: C, 29.32; H, 2.48; N, 68.2.

### Experimental

The thermal decompositions of **1** and **2** under flowing N_2_ were investigated using a thermogravimetric analyzer (Netzsch STA 449 C; Selb, Germany) and a differential scanning calorimeter (DSC Q2000; New Castle, USA). The TG conditions were as follows: sample mass, ca. 1.0 mg; heating rates, 5, 7, 10, 13, and 20 °C min^−1^ for **1**, and 5, 7, 10, 15, and 20 °C min^−1^ for **2**; atmosphere, N_2_ (flow rate 30 mL min^−1^); temperature range, 20–400 °C. The DSC conditions were as follows: heating rates, 5, 7, 10, 13, and 20 °C min^−1^ for **1**, and 5, 7, 10, 15, and 20 °C min^−1^ for **2**; atmosphere, N_2_ (flow rate 30 mL min^−1^); temperature range, 20–400 °C. All TG–DSC data were analyzed using Proteus Analysis software.

Thermal pyrolyses of **1** and **2** were investigated using a pyrolysis (EGA/PY-3030D, Fukushima-ken, Japan) gas chromatography–mass spectrometry (QP2010-Ultra, Kyoto, Japan) instrument (PY–GC/MS). The PY conditions were as follows: pyrolysis temperatures, 400 and 500 °C; pyrolysis time, 1 min; injection port temperature, 300 °C. The GC/MS conditions were as follows: capillary chromatographic column, ZB-5HT (30 mm × 0.25 mm × 0.25 μm); heating program: 50 °C, holding for 3 min, then heating to 300 °C at a rate of 10 K min^−1^, holding to 5 min; injection port temperature, 300 °C; split injection; split ratio 100:1; carrier gas (high-purity helium, 99.9999%) flow rate 1.0 mL min^−1^; collector temperature, 280 °C; ion source temperature, 250 °C; ion source scan mode, full scan (40–100 *m*/*z*).

### Model-free methods

By using these methods, the kinetic parameters of a solid-state reaction can be obtained without knowing the reaction mechanism.

In previous work [29–32], Kissinger method was widely used to determine the activation energies with the reaction process that occur under linear heating rate conditions. Although this method has some limitations, it is acceptable when an isoconversional method was used to back up the veracity of the Kissinger method [33]. In addition, since the decomposition reaction process of the title compounds would be very complex, the values of *E*_*α*_ of the title compounds obtained by the two isoconversional methods namely Kissinger–Akahira–Sunose [34] and Starink [35] method vary greatly and are disorder in the given range of α as 0.05–0.95. And many other types of kinetic methods [33] were tried to back up the veracity of the Kissinger method, but all of the results are unsatisfactorily. According to the literature [29, 36–39], an applicable method namely Ozawa–Doyle method was commonly used to back up the Kissinger method in the kinetic calculation of the energetic materials for its’ acceptable result.

#### Kissinger method

In 1957, Kissinger [40] first introduced a model-free non-isothermal method, which can help researchers to evaluate kinetic parameters without the need of calculating *E* for each conversion value of the solid-state reaction. This method is described as follows:

1 $$\ln \left( {\frac{\beta }{{T_{P}^{2} }}} \right) = \ln \frac{AR}{E} - \frac{E}{{RT_{P} }}$$

In this equation, *T*_*P*_ is the peak temperature of the DSC curve. The apparent activation energy (*E*) and pre-exponential factor (*A*) can be obtained from the slope −/(*RT*_*P*_) and intercept ln(*AR/E*) respectively, of an ln (*β/T*_*P*_^*2*^) versus 1/*T*_*P*_ plot.

#### Ozawa–Doyle method

The Ozawa–Doyle method [41, 42] is simple and applicable to reactions that cannot be analyzed by other methods. It has been widely used to determine the apparent activation energy (*E*) alongside the Kissinger method. The Ozawa–Doyle equation is as follows:

2 $$\log \beta + \frac{0.4567E}{{RT_{P} }} = C$$

where *T*_*P*_ is the peak temperature of the DSC curve and *C* is a constant. The apparent activation energy (*E*) can be obtained from a plot of logarithm of heating rates. A plot of log*β* versus 1/*T*_*P*_ expresses a linear function with an intercept of 0.4567*E/R*. The calculated *E* of this method is independent of the mechanism of thermal decomposition.

## Results and discussion

### Thermogravimetric analysis

**Fig. 2:**
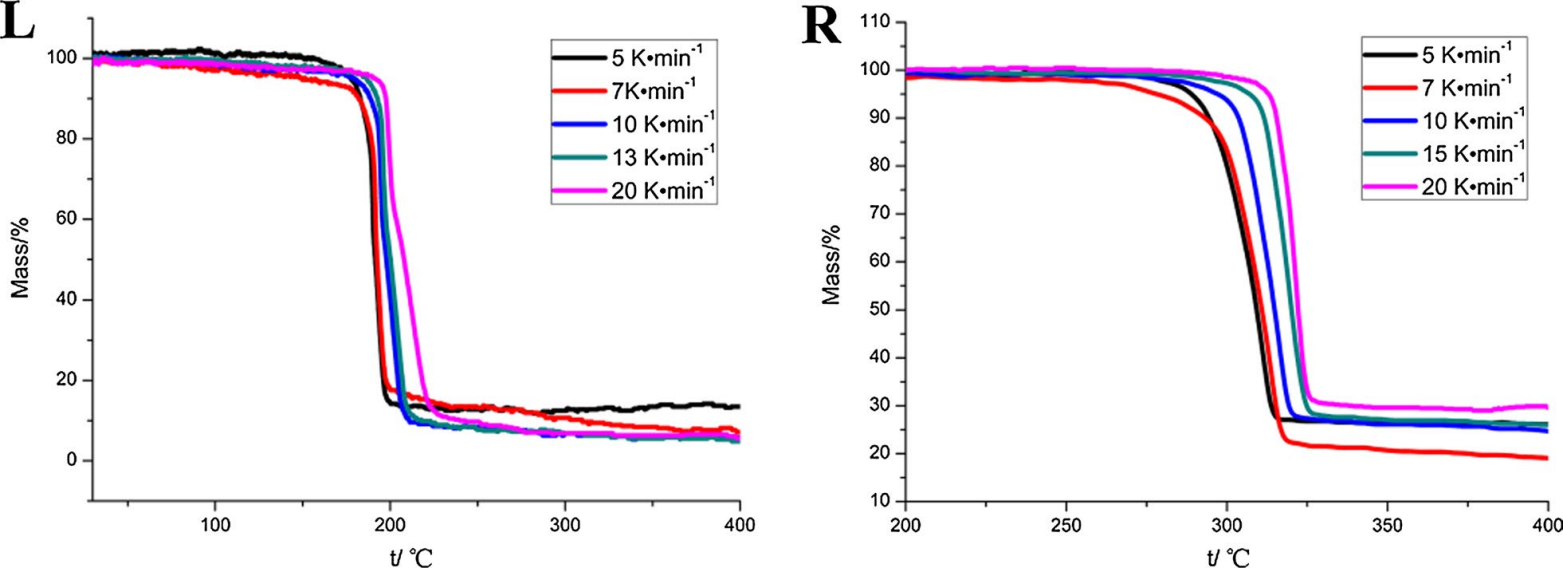
TG traces of compound **1** (L) and compound **2** (R) obtained at different heating rates

**Fig. 3:**
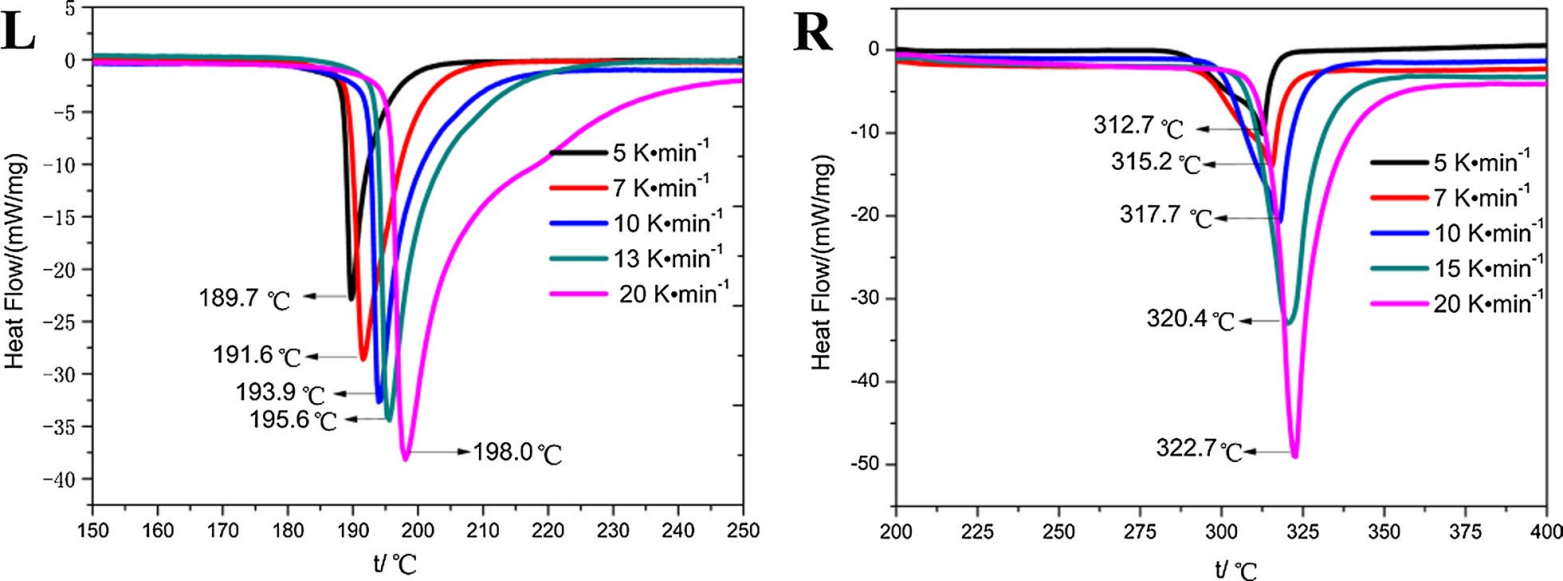
DSC traces of compound **1** (L) and compound **2** (R) obtained at different heating rates

The TG and DSC curves of **1** and **2** at different heating rates under N_2_ atmosphere are shown in Figs. 2 and 3, respectively. The DSC curves show that the thermal decompositions of **1** and **2** at different heating rates involve a single exothermic process. In this process, the molecules of **1** and **2** were broken into smaller pieces by cleavage of the N=N bond linking the two triazole rings and opening of their triazole rings. At the same time, the TG curves provided insight into the total weight losses of the compounds associated with this process.

Characteristic temperatures at different heating rates in the TG–DSC curves of **1** and **2** are shown in Table 2. From Table 2, it can be seen that at 5 °C min^−1^, thermal decompositions of **1** and **2** started at 176.1 and 281.7 °C, respectively. At higher heating rates, the initial temperature (*T*_*0*_), the extrapolated onset temperature (*T*_*e*_), and the peak temperature (*T*_*P*_) of the DSC curves shifted from 176.1, 188.5, and 189.7 °C at 5 °C min^−1^ to 189.8, 199.7, and 198.0 °C at 20 °C min^−1^, respectively, for **1**. For **2**, *T*_*0*_, *T*_*e*_, and *T*_*P*_ shifted from 281.7, 288.8, and 312.7 °C at 5 °C min^−1^ to 302.1, 315.1, and 322.7 °C at 20 °C min^−1^, respectively. These data show that with increased heating rate, the values of *T*_*0*_, *T*_*e*_, and *T*_*P*_ increase. This behavior can be attributed to heat transfer between the sample and the instrument.

**Table 2:** The characteristic temperatures of the title compounds at different heating rates and the kinetic parameters

1,1ʹ-Azobis-1,2,3-triazole				4,4ʹ-Azobis-1,2,4-triazole			
Heating rates *β*/°C min^−1^	*T*_*0*_/°C	*T*_*e*_/°C	*T*_*P*_/°C	Heating rates *β*/°C min^−1^	*T*_*0*_/°C	*T*_*e*_/°C	*T*_*P*_/°C
5	176.1	188.5	189.7	5	281.7	288.8	312.7
7	179.3	189.2	191.6	7	286.2	294.5	315.2
10	181.2	192.5	193.9	10	289.7	299.8	317.7
13	183.3	193.4	195.6	15	298.6	309.2	320.4
20	189.8	199.7	198.0	20	302.1	315.1	322.7
	*T*_*00*_/°C	*T*_*e0*_/°C	*T*_*P0*_/°C		*T*_*00*_/°C	*T*_*e0*_/°C	*T*_*P0*_/°C
	163.94	180.82	183.32		278.00	276.13	303.34

Data calculated by the Kissinger method							
*E*_*K*_/kJ mol^−1^	291.4	396.2					
ln(*A*_*K*_*/*s^−1^)	75.53	80.98					
Linear correlation coefficient (*r*_*K*_)	0.9986	0.9989					
Standard deviation	0.0185	0.0158					
Data calculated by the Ozawa–Doyle method							
*E*_*O*_/kJ mol^−1^	284.5	386.1					
Linear correlation coefficient (*r*_*O*_)	0.9986	0.9990					
Standard deviation	0.0181	0.0154					
*E*_*a*_/kJ mol^−1^	287.95	391.15					

*E*_*a*_ is the average of *E*_*K*_ and *E*_*O*_

### Kinetic analysis

From the thermogravimetric analysis results, we can calculate the kinetic parameters according to the model-free methods. The activation energy (*E*) and pre-exponential factor (*A*) were obtained using the Kissinger and Ozawa–Doyle methods.

From the original data of the exothermic peak temperature measured at five different heating rates of 5, 7, 10, 13, and 20 °C min^−1^ for **1**, and 5, 7, 10, 15, and 20 °C min^−1^ for **2**, the apparent activation energies *E*_*K*_ and *E*_*O*_, the pre-exponential factors *A*_*K*_, and the linear coefficients *r*_*K*_ and *r*_*O*_ were determined, as shown in Table 2.

From Table 2, it can be seen that the apparent activation energies (*E*) for **1** and **2** obtained by the Kissinger method are very close to the values obtained by the Ozawa–Doyle method. The minor differences are assumed to stem from limitations of the method itself and errors in calculation. Moreover, the absolute values of the linear correlation coefficients (*r*) for **1** and **2** in Table 2 are close to 1, which indicates that the kinetic parameters were obtained with high accuracy.

The Arrhenius equations of the title compounds can be expressed as follows (*E* is the average of *E*_*K*_ and *E*_*O*_):

3 $$\begin{aligned}\ln k &= 75.53 - 287.95 \times 10^{3} /\left( {RT} \right) \\ &\quad\quad\quad\quad{\text{ for }}1,1^{\prime}{\text{-azobis-}}1,2,3{\text{-triazole}} \end{aligned}$$

4 $$\begin{aligned}\ln k &= 80.98 - 391.15 \times 10^{3} /\left( {RT} \right)\\ &\quad\quad\quad\quad{\text{ for }}4,4^{\prime}{\text{-azobis-}}1,2,4{\text{-triazole}} \end{aligned}$$

The values of *T*_*00*_, *T*_*e0*_, and *T*_*P0*_ corresponding to *β* → 0 obtained from Eq. (5) are shown in Table 2.

5 $$\begin{aligned}T_{{\left( {0,e,p} \right)i}} = T_{{\left( {00,e0,p0} \right)i}} + b\beta_{{_{i} }} + c\beta_{i}^{2} + d\beta_{i}^{3} \\ &\quad\quad\quad\quad i = 1,2,3,4,5 \end{aligned}$$

where *b*, *c*, and *d* are coefficients.

The critical temperatures of thermal explosion (*T*_*b*_) were obtained according to Eq. (6) as 187.01 °C for **1** and 282.78 °C for **2**, respectively [43, 44].

6 $$T_{b} = \frac{{E_{O} - \sqrt {E_{O}^{2} - 4E_{O} RT_{e0} } }}{2R}$$

where *E*_*O*_ is the value of *E* obtained by the Ozawa–Doyle method.

Evidently, the values of apparent activation energy (*E*), extrapolated onset temperature (*T*_*e*_), and critical temperature of thermal explosion (*T*_*b*_) for **2** are consistently higher than those for **1**, indicating greater thermodynamic stability of the former. Comparing the values of *T*_*b*_ with those for other common energetic compounds: CL-20 (202.07 °C), HMX (267.43 °C), RDX (209.32 °C), NTO (265.53 °C), ENTO (227.44 °C), KNTO (226.32 °C) [33], ZTO (282.21 °C), ATO (299.64 °C), GZTO**·**H_2_O (237.74 °C) [45], and KZTO·H_2_O (275.08 °C) [30], the thermodynamic stability sequence of these compounds can be expressed as: **1** < CL-20 < RDX < KNTO ≈ ENTO < GZTO·H_2_O < NTO ≈ HMX < KZTO·H_2_O < ZTO ≈ **2** < ATO.

### Thermal pyrolysis analysis

Pyrolysis–gas chromatography–mass spectrometry (PY–GC/MS) can be used to qualitatively analyze pyrolysis products. The pyrolysis chamber was heated to the preset temperature, then the sample was added, after 3 s, a fast heating process was performed and the pyrolysis process was carried out. Fragments were then separated by the GC column and their structures were identified by the MS system. For unknown compounds, one can obtain important information, such as their composition, microstructure, and so on. For known compounds, one can determine the pyrolysis products, and thereby infer the pyrolysis reaction pathways of the compound. This method has many advantages, including a very small injection volume, suitability for a broad range of samples, rapid analysis, and good reproducibility.

Because the molecules of **1** and **2** contain many C–N/N–N single and double bonds, their critical temperatures of thermal explosion (*T*_*b*_) are below 300 °C. Hence, the explosion reaction must happen during the pyrolysis process, and the actual temperature at the reaction center may briefly reach thousands of degrees Celsius. In the pyrolysis of **1** and **2**, following the initial decomposition of the reactive molecule, the pyrolysis products could undergo a multitude of collisions and numerous other reaction processes prior to collection and analysis by the GC/MS system. Hence, investigation of the rapid explosion reaction is a very difficult task, and the mechanistic interpretation inferred by us in this work should be placed in the context of the difficulty in truly isolating microscopic pathways of the explosion reaction.

**Fig. 4:**
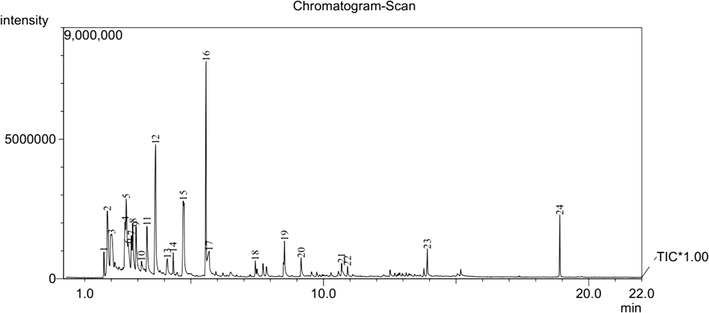
PY–GC/MS total ion chromatogram of 1,1ʹ-azobis-1,2,3-triazole at 400 °C

**Fig. 5:**
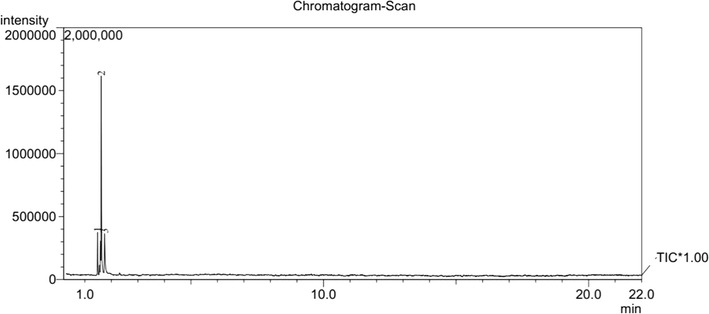
PY–GC/MS total ion chromatogram of 1,1ʹ-azobis-1,2,3-triazole at 500 °C

**Fig. 6:**
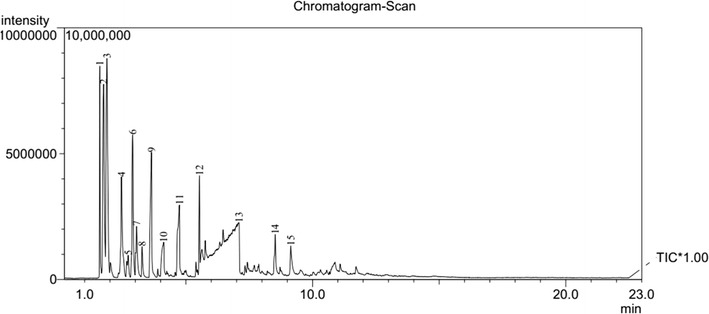
PY–GC/MS total ion chromatogram of 4,4ʹ-azobis-1,2,4-triazole at 400 °C

**Fig. 7:**
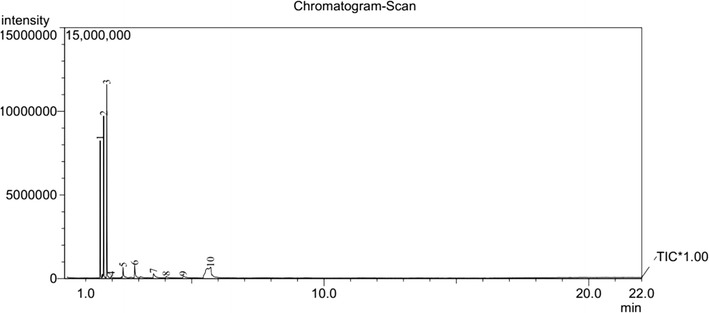
PY–GC/MS total ion chromatogram of 4,4ʹ-azobis-1,2,4-triazole at 500 °C

The pyrolysis–mass spectrometric traces of **1** and **2** at 400 and 500 °C are shown in Figs. 4, 5, 6, 7, respectively. From these figures, it can be seen that for different pyrolysis temperatures, the compositions and types of the pyrolysis products differ greatly. At 400 °C, the retention times of the first three pyrolysis products into the chromatogram were similar to those at 500 °C, but the number of pyrolysis products was fewer at 500 °C, indicating that the pyrolysis reaction at 500 °C is more thorough than 400 °C.

We could estimate the relative contents of the pyrolysis products from a qualitative comparison of the masses of all components using the peak area normalization method. The structures and the relative contents of the pyrolysis products from **1** and **2** are shown in Tables 3 and 4, respectively.

**Table 3:**
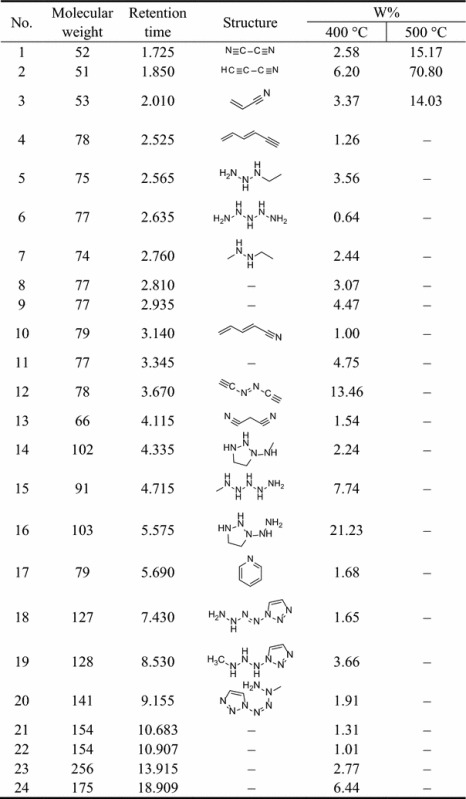
Pyrolysis spectral peaks and product structural assignments for 1,1′-azobis-1,2,3-triazole

**Table 4:**
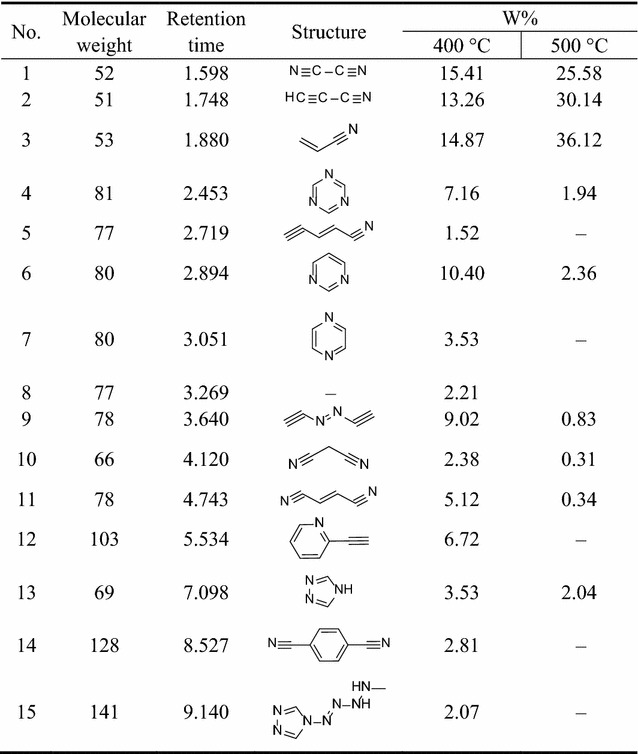
Pyrolysis spectral peaks and product structural assignments for 4,4ʹ-azobis-1,2,4-triazole

**Fig. 8:**
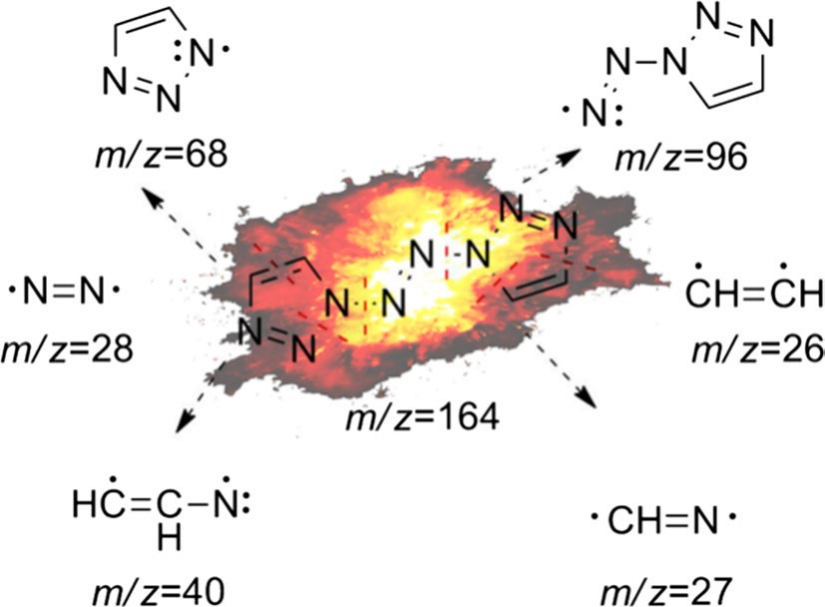
The explosion reaction of 1,1ʹ-azobis-1,2,3-triazole at the pyrolysis temperature

From Table 3, it is clear that with increasing pyrolysis temperature, the numbers of different pyrolysis products were significantly reduced, and the structures were also much simpler. At 400 °C, there were about 24 species among the pyrolysis products of **1**, and the differences between the proportions of the different types of pyrolysis products were relatively large. From the pyrolysis results, it is clear that it is difficult to create all of the fragments through direct cleavage of the starting molecules. Hence, we assume that in the explosion reaction, numerous molecules extensively broken into small fragment ions, as shown in Fig. 8, and these fragments underwent secondary reactions, such as coupling, rearrangement, addition, and elimination of hydrogen atoms, prior to detection by the GC/MS system.

**Fig. 9:**
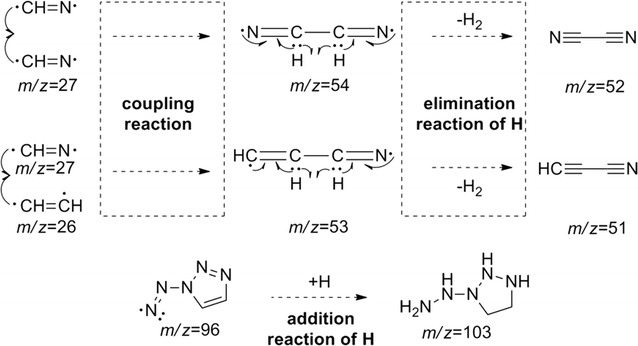
Several secondary reactions of the initial fragment ions to form the observed fragments

The earliest species observed in the pyrolysis at 400 °C were those with *m*/*z* 52, *m*/*z* 51, and *m*/*z* 53, and these species were the sole products at 500 °C. Among these, that at *m*/*z* 52 may have arisen from coupling of two *m*/*z* 27 (CH=N) fragments by C–C single-bond formed with the elimination of two hydrogen atoms. In the same way, the fragments with *m*/*z* 51 and *m*/*z* 53 could have been created from that of *m*/*z* 26 (CH=CH) coupling with that of *m*/*z* 27 (CH=N) through the formation of a C–C single bond, the former with the concurrent elimination reaction of two hydrogen atoms. The larger molecular mass of 103 may have been created by hydrogenation of the fragment with *m*/*z* 96. All of the other fragment ions can reasonably be formed from the small fragment ions (as shown in Fig. 8) originating from the explosion reaction of **1**, through coupling and rearrangement reactions, sometimes with the concurrent addition or elimination of hydrogen atoms, as shown in Fig. 9.

**Fig. 10:**
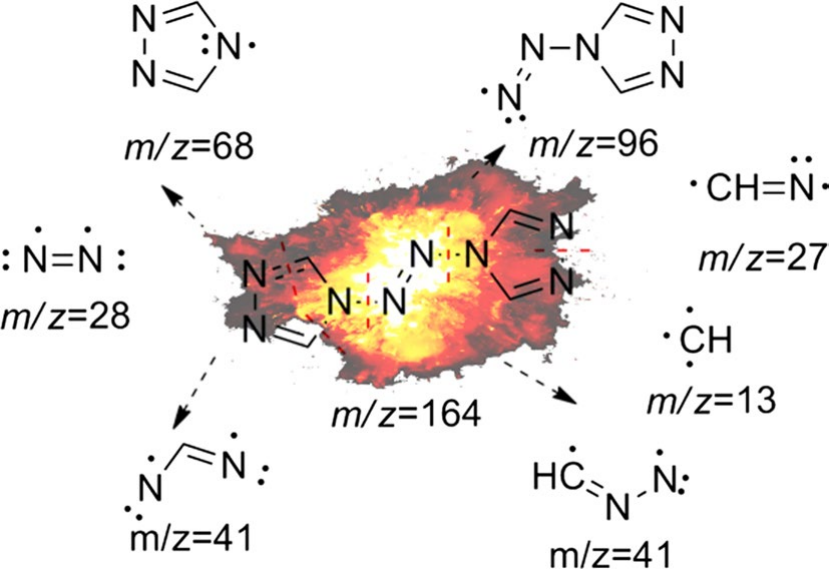
The explosion reaction of 4,4ʹ-azobis-1,2,4-triazole at the pyrolysis temperature

From Table 4, the same conclusion can be reached. In the explosion reaction process of **2**, numerous molecules were extensively broken into much smaller fragment ions, as shown in Fig. 10. In the same way, these fragments could undergo a multitude of secondary reactions prior to detection by the GC/MS system.

**Fig. 11:**
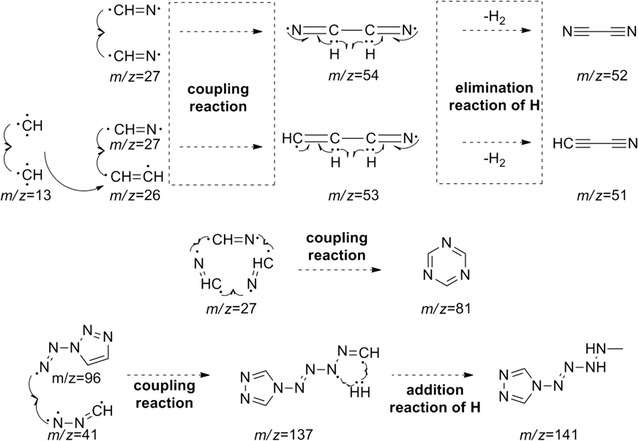
Several secondary reactions of the initial fragment ions to form the observed fragments

The principal pyrolysis products of **2** were also those with *m*/*z* 52, *m*/*z* 51, and *m*/*z* 53, where that at *m*/*z* 52 can be created by coupling of two fragments with *m*/*z* 27 (CH=N) through C–C single-bond formation with the elimination of two hydrogen atoms. In the same way, those at *m*/*z* 51 and *m*/*z* 53 can be created by coupling of two fragments with *m*/*z* 13 (CH) through C=C double-bond formation creating a fragment with *m*/*z* 26 (HC=CH), followed by coupling with a fragment of *m*/*z* 27 (CH=N) through formation of a C–C single bond, the former with the concurrent elimination of two hydrogen atoms. The fragment with *m*/*z* 81 may have been created as a trimer of that with *m*/*z* 27 (CH=N), and that at *m*/*z* 141 may have been created by a fragment with *m*/*z* 96 coupling with a fragment with *m*/*z* 41 (N–N=CH) and the addition of hydrogen atoms, as shown in Fig. 11.

Nevertheless, some fragments are yet unaccounted for, such as those with *m*/*z* 77 (Table 3, entries 8, 9, 11), *m*/*z* 154 (entries 21, 22), *m*/*z* 256 (entry 23), and *m*/*z* 175 (entry 24) for **1**, and *m*/*z* 77 (Table 4, entry 8) for **2**.

## Conclusion

Experimental kinetic studies on the thermal decomposition processes of two typical nitrogen-rich energetic materials (**1** and **2**) were described, in which kinetic parameters, namely the apparent activation energy (*E*) and pre-exponential factor (ln*A*), were determined by the Kissinger and Ozawa–Doyle methods.

By the Kissinger method, values of *E* as 291.4 and 396.2 kJ mol^−1^ were obtained for **1** and **2**, respectively, with ln*A*(s^−1^) values of 75.53 and 80.98, respectively. By the Ozawa–Doyle method, the values of *E* were 284.5 and 386.1 kJ mol^−1^ for **1** and **2**, respectively, showing good agreement. The linear correlation coefficients (*r*) were close to 1, validating the results.

The critical temperatures of thermal explosion (*T*_*b*_) were determined as 187.01 °C for **1** and 282.78 °C for **2**. From the values of *E*, *A*, and *T*_*b*_, **2** is clearly more thermodynamically stable than **1**. Critical temperatures of thermal explosion follow the sequence: **1** < CL-20 < RDX < KNTO ≈ ENTO < GZTO·H_2_O < NTO ≈ HMX < KZTO·H_2_O < ZTO ≈ **2** < ATO.

By PY–GC/MS, thermal pyrolyses of **1** and **2** at 400 and 500 °C generated a greater number of species. By analysis of the possible structures of the pyrolysis products, some conclusions about the pyrolysis pathways of **1** and **2** were drawn. The fragments detected by GC/MS following the pyrolyses of **1** and **2** were likely due to numerous secondary reactions, such as coupling, rearrangement, and addition or elimination of hydrogen atoms, of the smaller ion fragments derived from the explosion reactions (Additional file 1).

## Additional file

**Additional file 1.** The purity of the title compounds.
