# Supplementary material for: Thermogravimetric analysis, kinetic study, and pyrolysis–GC/MS analysis of 1,1ʹ-azobis-1,2,3-triazole and 4,4ʹ-azobis-1,2,4-triazole
Source: Chem Cent J. 2018 Mar 1;12:22. doi: 10.1186/s13065-018-0381-x (PMC5833888; doi:10.1186/s13065-018-0381-x)
Supplement: Supplementary file 1 — Additional file 1. The purity of the title compounds. [file 13065_2018_381_MOESM1_ESM.docx]

Supporting information for

**Thermogravimetric Analysis, Kinetic Study, and Pyrolysis-GC/MS Analysis of 1,1ʹ-Azobis-1,2,3-triazole and 4,4ʹ-Azobis-1,2,4-triazole**

Chenhui Jia,^a^ Yuchuan Li,*^a^ Shujuan Zhang,^a^ Teng Fei^a^ and Siping Pang^a^

School of Material Science & Technology, Beijing Institute of Technology, Beijing 100081, P.R. China*,* Email: [**liyuchuan@bit.edu.cn**](mailto:liyuchuan@bit.edu.cn)

**Contents of the supporting information:**

The purity of the title compounds S1

**S1**

**The purity of the title compounds**

The purity of the title compounds was determined using an Agilent 1260 HPLC equipped with an Eclipse XDB C18 chromatographic column. The temperature was held at 303.15 K during the analytical procedure. The wavelength of the ultraviolet detector was set to 230 nm and the sample injection volume was 5 μL. The mobile phase was methanol/water (90/10), injected at a flow rate of 0.4 mL·min^−1^. The results were shown in Figure 2 and 3.


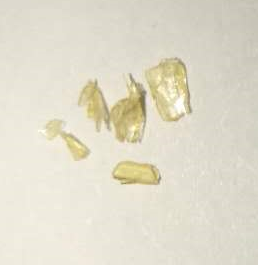

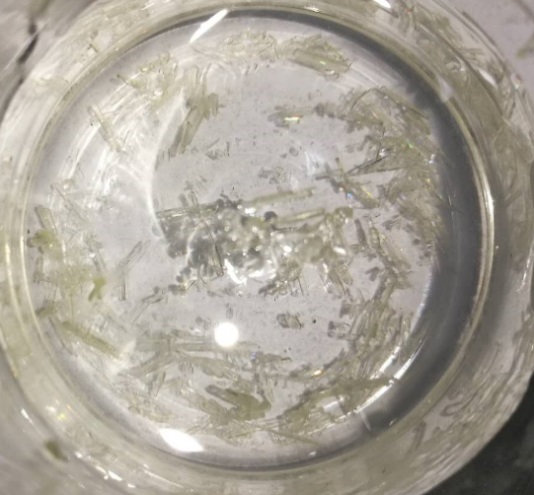


**4,4’-azobis-1,2,4-tirazole**

**1,1’-azobis-1,2,3-tirazole**

**Fig.1** The crystal of 1,1ʹ-azobis-1,2,3-triazole (**1**) and 4,4ʹ-azobis-1,2,4-triazole (**2**)


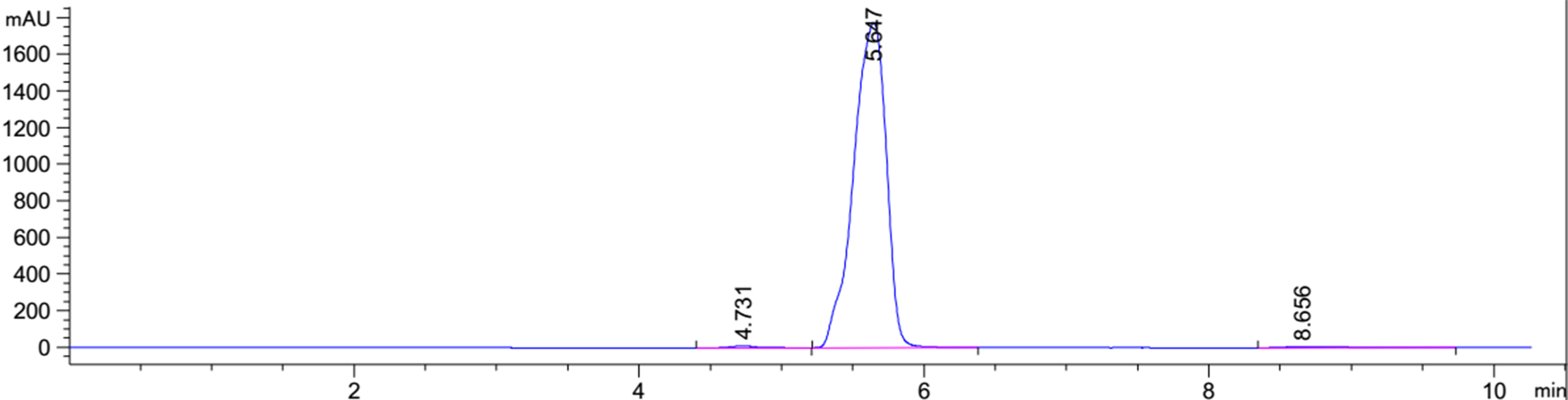


**Fig.2** High-performance liquid chromatograph (HLPC) of **1**


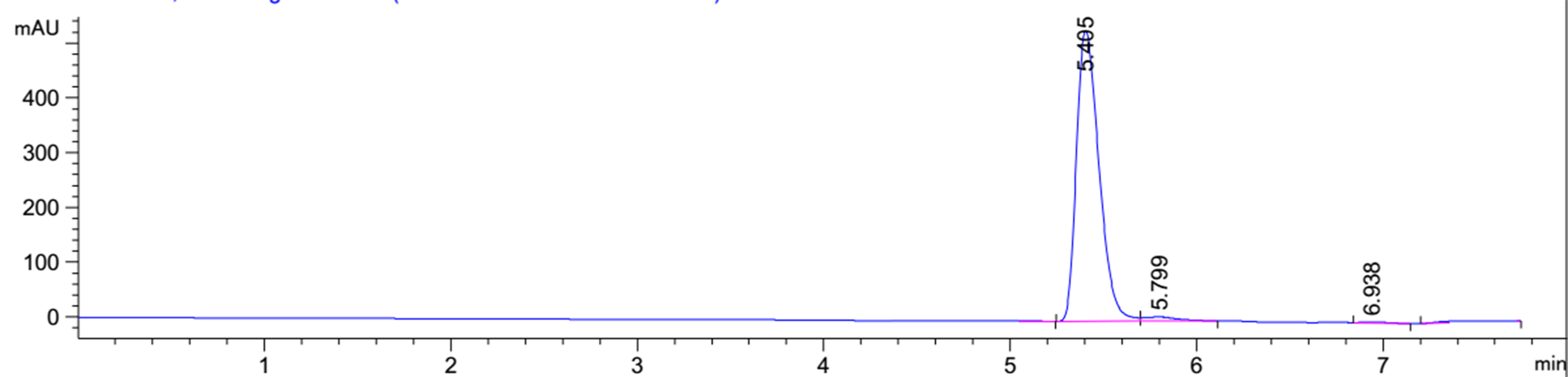


**Fig.3** High-performance liquid chromatograph (HLPC) of **2**
